# ^1^H-NMR-Based Metabolomic Study of Tomato Cultivars, Heinz and Roma, Grown on Selected Growth Medium and Deep-Water Hydroponic Culture System

**DOI:** 10.3390/metabo16090624

**Published:** 2026-08-28

**Authors:** Sinenhlanhla Nonhle Nsele, Maropeng Vellry Raletsena, Udoka Vitus Ogugua, Pierre Adriaanse

**Affiliations:** 1 Department of Agriculture and Animal Health, College of Agriculture and Environmental Sciences, University of South Africa, Florida 1710, South Africa; 49718037@mylife.unisa.ac.za; 2 Laboratories and Horticulture Centre, Science Campus, University of South Africa, Florida 1710, South Africa; raletmv@unisa.ac.za (M.V.R.); adriap@unisa.ac.za (P.A.)

**Keywords:** nuclear magnetic resonance, principal component analysis, coconut coir, peat moss, deep-water culture

## Abstract

**Background/Objectives:** This study examined the effect of three different cultural media—coconut coir, peat moss, and deep-water culture—on the metabolomic profiles of two tomato cultivars, Heinz and Roma. Tomato fruit quality is influenced by intricate interactions between the genotype and root-zone environment; nevertheless, little is known about how substrate-based systems compare metabolically to deep-water hydroponics. **Methods:** Fruit samples from both cultivars grown under controlled greenhouse conditions were analyzed using proton nuclear magnetic resonance (^1^H-NMR) spectroscopy to identify treatment-dependent biochemical changes. Polar metabolites were extracted using a methanol–water solvent solution and examined using a 600 MHz NMR spectrometer. Spectral datasets were processed and analyzed with multivariate statistical tools such as Principal Component Analysis (PCA), Partial Least Squares Discriminant Analysis (PLS-DA), and Orthogonal Partial Least Squares Discriminant Analysis (OPLS-DA). **Results:** Distinct clustering patterns were observed, indicating both cultivar-specific and cultivation-system-dependent metabolic differentiation. The PCA model displayed excellent explanatory and predictive capacity, while supervised OPLS-DA improved group discrimination, demonstrating that both genotype and growing medium significantly influenced the chemical composition of the fruit. Soluble sugars (glucose, fructose, and sucrose), sugar alcohols, organic acids such as citric and malic acids, and a variety of amino acids involved in nitrogen metabolism and stress reactions were among the key distinguishing factors. Carbohydrate-related spectral areas (3.0–5.5 ppm) were highly associated with treatment separation, indicating that the cultivation system had a significant impact on carbon allocation and energy metabolism. **Conclusions:** Fruits grown in deep water culture have distinct metabolic fingerprints from those grown on coconut coir and peat moss, implying that root-zone oxygen availability and nutrient dynamics may influence primary and secondary metabolism.

## 1. Introduction

Tomato (*Solanum lycopersicum* L.) is one of the most widely consumed vegetables globally, valued for its nutritional content, including vitamin C, folate, carotenoids, phenolic compounds, and lycopene, which contributes to its well-established health benefits, such as reduced risk of cardiovascular disease and certain cancers [1]. Its short life cycle and well-characterized genetics have also made tomatoes an important model organism in studies of plant physiology, metabolism, and environmental responses [2]. The metabolic complexity of tomato fruit, combined with increasing interest in its nutritional and organoleptic attributes, has elevated the importance of metabolomics in understanding how cultivar differences and cultivation conditions shape fruit quality [3,4].

Among the analytical tools, nuclear magnetic resonance (^1^H-NMR) spectroscopy has become a powerful platform for plant metabolomics because of its high reproducibility, minimal sample preparation, and ability to simultaneously quantify a wide range of metabolites. Studies analyzing tomato fruit using ^1^H-NMR have successfully identified sugars, amino acids, organic acids, and other key metabolites, demonstrating clear metabolomic distinctions among cultivars and tissue types. Additionally, when combined with multivariate statistical techniques, NMR-based metabolomics has demonstrated tremendous benefit in understanding the biochemical network dynamics in tomatoes [3,5].

Cultivation practices, including substrate-based and hydroponic systems, significantly influence the metabolic composition of tomato. Hydroponic techniques, especially deep-water culture (DWC), enable tighter control of nutrient delivery, water availability, and root-zone conditions, often resulting in altered fruit quality attributes. Controlled comparisons between soil and hydroponic systems have shown that hydroponically grown tomatoes can exhibit higher water-use efficiency and elevated levels of metabolites such as lycopene and β-carotene, highlighting the potential of hydroponic cultivation to modulate metabolic outcomes. These metabolic shifts underscore the importance of linking cultivation conditions with biochemical profiles to optimize both yield and fruit quality [6].

Despite extensive research on tomato metabolomics, comparative ^1^H-NMR-based assessments focusing specifically on commercially important cultivars such as Heinz and Roma grown under different cultivation systems remain limited. Understanding how these cultivars respond metabolically to selected growth media versus deep-water hydroponic systems is crucial for identifying cultivation-dependent metabolic signatures that influence nutritional, sensory, and processing quality traits. Building on the strengths of NMR metabolomics and the increasing adoption of hydroponic systems, the present study aimed to advance this knowledge by characterizing and comparing the metabolomic profiles of Heinz and Roma tomatoes cultivated under controlled substrate-based (coconut coir and peat moss) and deep-water culture conditions.

## 2. Materials and Methods

### 2.1. Description of Study Site and Plant Materials Used

The experiment was conducted in a greenhouse at the University of South Africa (UNISA) Florida Campus in Johannesburg, Gauteng Province, South Africa (26°09.501′ S, 27°54.113′ E). An automated climate control system maintained greenhouse temperatures between 28 and 30 °C, with a 12 h light-dark photoperiod to replicate natural diurnal settings. The temperature and humidity were controlled using ventilation fans and an evaporative cooling system, which provided appropriate air movement while reducing heat accumulation. Standard greenhouse management methods were followed throughout the trial. Seeds of the tomato cultivars Heinz and Roma were sown in seedling trays and grown under controlled greenhouse conditions. Seedlings were examined daily, and irrigation was provided manually as needed to maintain optimal moisture levels. After emergence, only strong and consistently growing seedlings were chosen for additional transplanting.

### 2.2. Growth Media Materials and Experimental Procedure

The experiment used a 2 × 3 factorial arrangement in a completely randomized design (CRD) with two tomato cultivars, Heinz and Roma, and three growing systems: coconut coir, peat moss, and deep-water culture (DWC), resulting in six treatment combinations (Table 1). The chemical composition and physicochemical characteristics of the growth substrates and nutrient solution are summarized in Table 2. At the four-leaf growth stage, the seedlings were transplanted into three systems. The solid growth media were coconut coir and peat moss, whereas the DWC system consisted of open containers filled with nutrient solutions and covered with perforated polystyrene trays that supported the seedlings. This design kept the plant roots submerged in the nutrient solution.

Each treatment included seven pots filled with the appropriate solid substrates, with three plants established in each. Each pot served as an experimental unit, with seven replicates per treatment (*n* = 7). To ensure consistency among the cultivation systems, the DWC setup was designed to mimic the experimental structure used for the solid medium. Peat moss was used as the control substrate. Within each growth system, 44 plants were produced, with an equal number of Heinz and Roma cultivars produced.

Fruit, leaf, and stem samples were collected at harvest maturity from each treatment combination for metabolomic analysis. Seven biological replicates were obtained for each of the six treatment combinations (Heinz–coconut coir, Roma–coconut coir, Heinz–peat moss, Roma–peat moss, Heinz–DWC, and Roma–DWC), resulting in a total of 42 biological samples for analysis. Fruit tissue from a separate experimental unit was collected and processed independently for ^1^H-NMR analysis in each biological replicate.

### 2.3. Growth Media, Nutrient Composition, and Experimental Design

This study examined the effects of three cultivation media (coconut coir, peat moss, and deep-water culture) on the metabolomic profiles of two tomato cultivars (Heinz and Roma). The experimental treatments included all possible cultivar and growth medium combinations: Heinz–coconut coir, Roma–coconut coir, Heinz–peat moss, Roma–peat moss, Heinz–DWC, and Roma–DWC. All treatments were subjected to uniform greenhouse management procedures to ensure that any identified changes in the metabolite profiles were attributable to the cultivation system and not environmental variation.

Coconut coir and peat moss served mostly as physical substrates, providing structural support for root development while contributing little to intrinsic nutrients. In contrast, the DWC system depended solely on a liquid nutritional formulation (Nitrosol Organiksol) to provide the required macro- and micronutrients. Table 1 summarizes the chemical composition and selected physicochemical features of the growth substrates and nutrient solutions used in this experiment.

### 2.4. Nutrient Management and Crop Maintenance

Plants cultivated in coconut coir, peat moss, and DWC systems were provided with the Nitrosol Organiksol nutrient solution to meet their nutritional needs (Table 2). The nutrient solution was applied at a concentration of 5 mL L^−1^ of water according to the manufacturer’s guidelines. Liu et al. [7] reported similar nutrient solution management systems for hydroponically cultivated tomatoes.

In the DWC system, the fertilizer solution was always available within the root zone, providing constant nutrient uptake. Continuous nutrient availability in hydroponic systems is critical for plant growth and productivity [8]. In the solid substrates (coconut coir and peat moss), the nutrient solution was manually applied at regular intervals and adjusted based on the plant growth stage and visual vigour assessment [9]. Substrate-based fertigation management strategies for coir-grown tomatoes were as described. Nutrient supply was maintained throughout the vegetative, flowering, and fruiting stages of the experiment. In the DWC system, the nutrient solution remained continuously available in the root zone, whereas in the solid substrates it was delivered manually at regular intervals, adjusted according to plant growth stage and visual assessment of plant vigour.

Uniform crop management practices were implemented across all treatments. These include routine monitoring of plant health, removal of senescent or damaged tissues when necessary, and regular inspection for nutrient deficiency symptoms. Greenhouse environmental conditions were consistent across treatments to minimize variability unrelated to the imposed cultivation systems.

### 2.5. Metabolomic Analysis

#### 2.5.1. NMR Sample Extraction

Proton nuclear magnetic resonance (^1^H NMR) spectroscopy was used for untargeted metabolomic profiling because of its high reproducibility and ability to simultaneously detect a wide range of primary and secondary metabolites. Metabolites were extracted using a methanol–water solvent system following established plant metabolomics protocols [10,11,12]. Frozen tomato fruit samples from the Deep-Water Culture–Heinz (DH), Deep-Water Culture–Roma (DR), coconut coir–Heinz (CH), and coconut coir–Roma (CR) treatments were finely pulverized into a homogeneous powder using a hammermill. Each sample (50 mg) was precisely weighed into 2 mL Eppendorf tubes for metabolite extraction.

For the extraction of each sample, 0.75 mL of deuterated methanol (CD_3_OD) and 0.75 mL of potassium dihydrogen phosphate (KH_2_PO_4_) buffer were generated in deuterium oxide (D_2_O; pH 6.0) with 0.01% (*w*/*w*) trimethylsilyl propionic acid (TSP) as the internal chemical shift reference. The mixtures were vortex-mixed at room temperature for 1 min, ultrasonically treated for 20 min to improve metabolite extraction, and centrifuged for 20 min at 10,000 rpm. The resultant supernatants were carefully transferred into 5 mm NMR tubes (Norell^®^, Sigma-Aldrich, St. Louis, MO, USA) before spectral capture.

#### 2.5.2. NMR Data Acquisition

A 600 MHz NMR spectrometer (Varian Inc., Palo Alto, CA, USA) was used to acquire the ^1^H NMR spectra. For each sample, 32 images were captured over a 20 ppm spectral width, resulting in an acquisition duration of approximately 7 min per spectrum. All spectra were subjected to uniform automated phasing and baseline adjustments using the MNova software (version 9.0.1, Mestre Lab Research, Santiago de Compostela, Spain). To eliminate interference, the spectra of residual water (δ 4.70–4.90 ppm) and methanol (δ 3.23–3.36 ppm) signals were excluded. Gradient shimming was used before acquisition to improve the magnetic field uniformity and spectral resolution.

#### 2.5.3. Data Mining and Processing of NMR Samples

The NMR spectra were bucketed using MestReNova software (9.0.1, Mestrelab Research, Santiago, Spain). The NMR areas were separated into 0.04 ppm bins. Principal component analysis (PCA) (unsupervised) and orthogonal partial least squares discriminatory analysis (OPLS-DA) (supervised) were used to conduct multivariate data analysis (MVDA). This was performed using Pareto scaling in SIMCA software (13.0, Umetrics, Malmö, Sweden). Chenomx commercial software and previously published NMR data were used to annotate the compounds. To visualize relative levels, heat maps based on the Pearson distance measure and Ward clustering technique were used to show the top metabolites for the control versus the treatments. To show important metabolites among the groups under study, multivariate tests, including principal component analysis (PCA), orthogonal partial least-squares discriminant analysis (OPLS-DA), and Partial Least Squares Discriminant Analysis (PLS-DA), were employed. One supervised technique for evaluating large data sets is OPLS-DA. Dendrogram analysis was used to identify the connections between metabolites. The main metabolites were identified using OPLS-DA and PLS-DA.

#### 2.5.4. Multivariate and Statistical Analysis

For multivariate statistical analysis, the SIMCA-P program (Version 13.0, Umetrics, Sweden) was used to import the processed ^1^1H-NMR spectral data. To reduce concentration differences between samples, spectral data were normalized to total spectral area prior to analysis. To increase the contribution of metabolites with moderate variance while lowering noise, the data were Pareto-scaled and mean-centered.

Initially, Principal Component Analysis (PCA) was used as an unsupervised exploratory technique to find potential outliers, trends, and intrinsic clustering patterns in the dataset. To maximize class discrimination among cultivars and cultivation treatments, partial least squares discriminant analysis (PLS-DA) and orthogonal partial least squares discriminant analysis (OPLS-DA) were subsequently used as supervised techniques.

**Figure 1:**
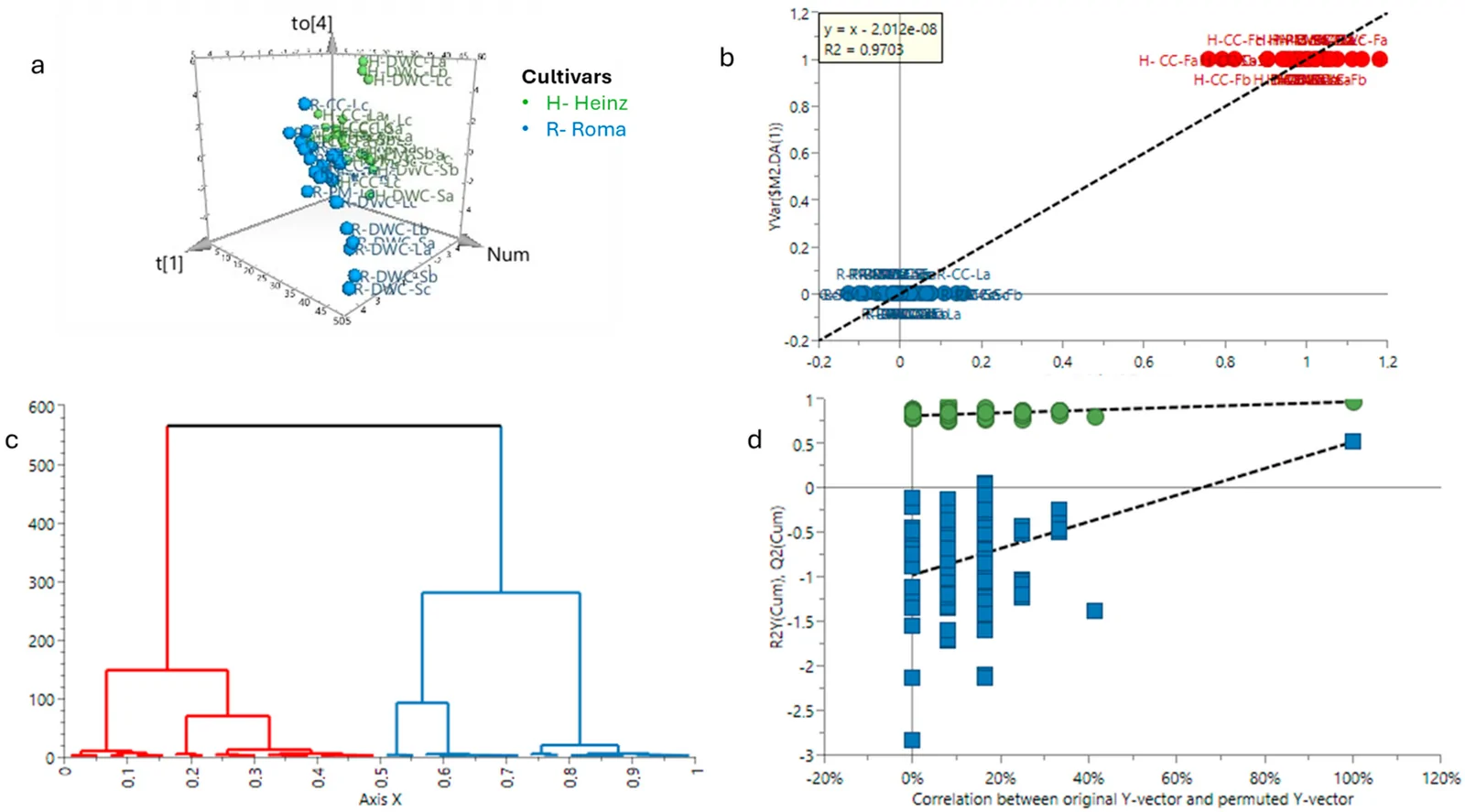
OPLS-DA and PLS-DA analyses of Heinz and Roma tomato cultivars grown under three cultivation treatments. (**a**) Three-dimensional (3D) score plot showing sample separation; (**b**) observed versus predicted score plot; (**c**) loading plot; and (**d**) permutation test plot used for model validation.

The predictive ability (Q^2^) and cumulative explained variance (R^2^X and R^2^Y) produced by seven-fold cross-validation were used to evaluate the quality of the model. Loading and contribution plots were used to identify variables that contribute to sample discrimination. Permutation testing (*n* = 200 permutations) and CV-ANOVA, when appropriate, were used to assess the supervised models’ robustness and significance. When CV-ANOVA results were statistically significant (*p* < 0.05) and Q^2^ values remained significantly higher than those from permuted models, the models were deemed valid. These validation techniques were employed to evaluate the model’s dependability and reduce the possibility of overfitting (Figure 1d).

## 3. Results

**Figure 2:**
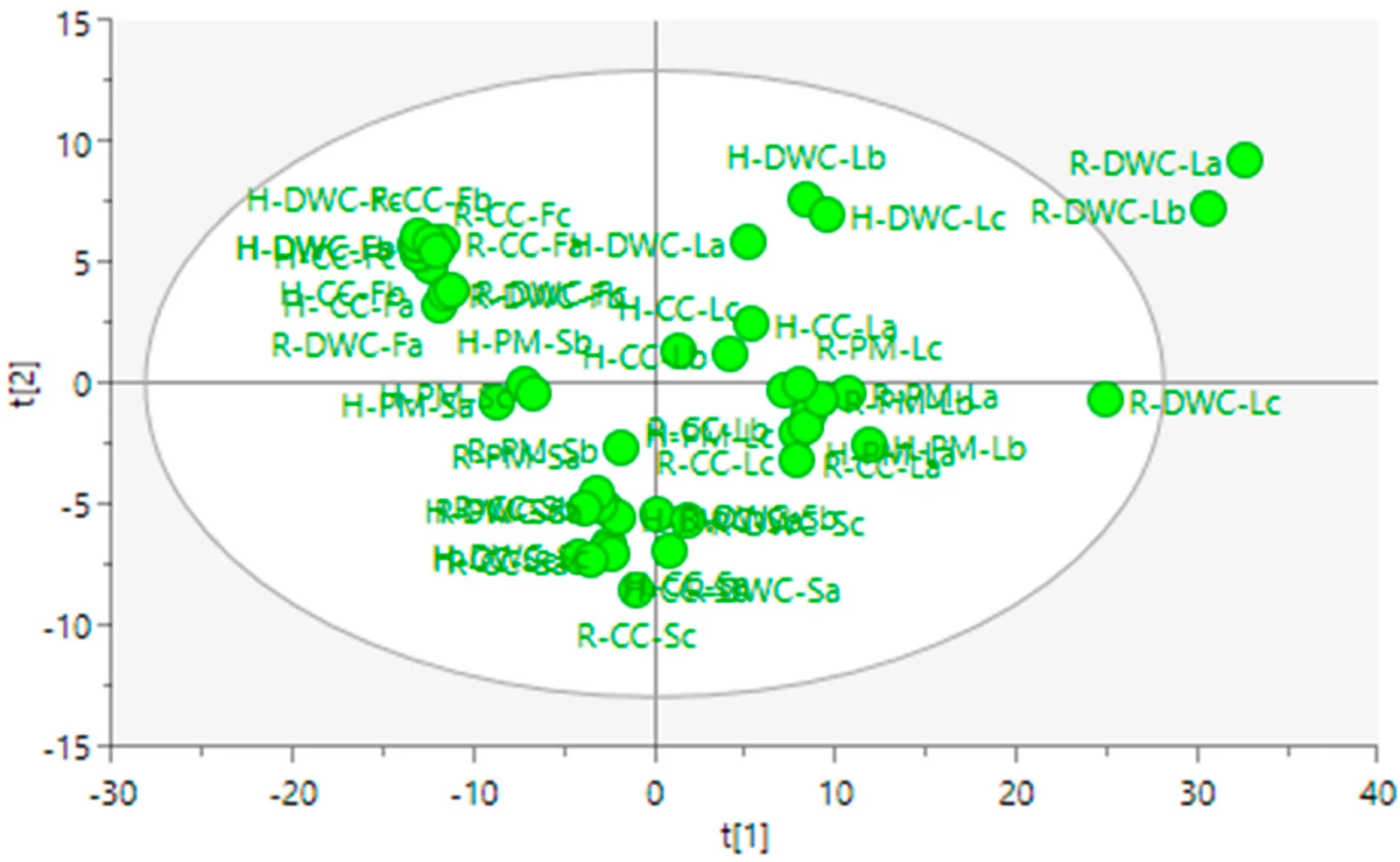
PCA-X score plot of two tomato cultivars (Heinz and Roma) grown under coconut coir (CC), peat moss (PM), and deep-water culture (DWC) systems. The PCA-X model provides an overview of the measured metabolite profiles and the clustering patterns among samples.

**Figure 3:**
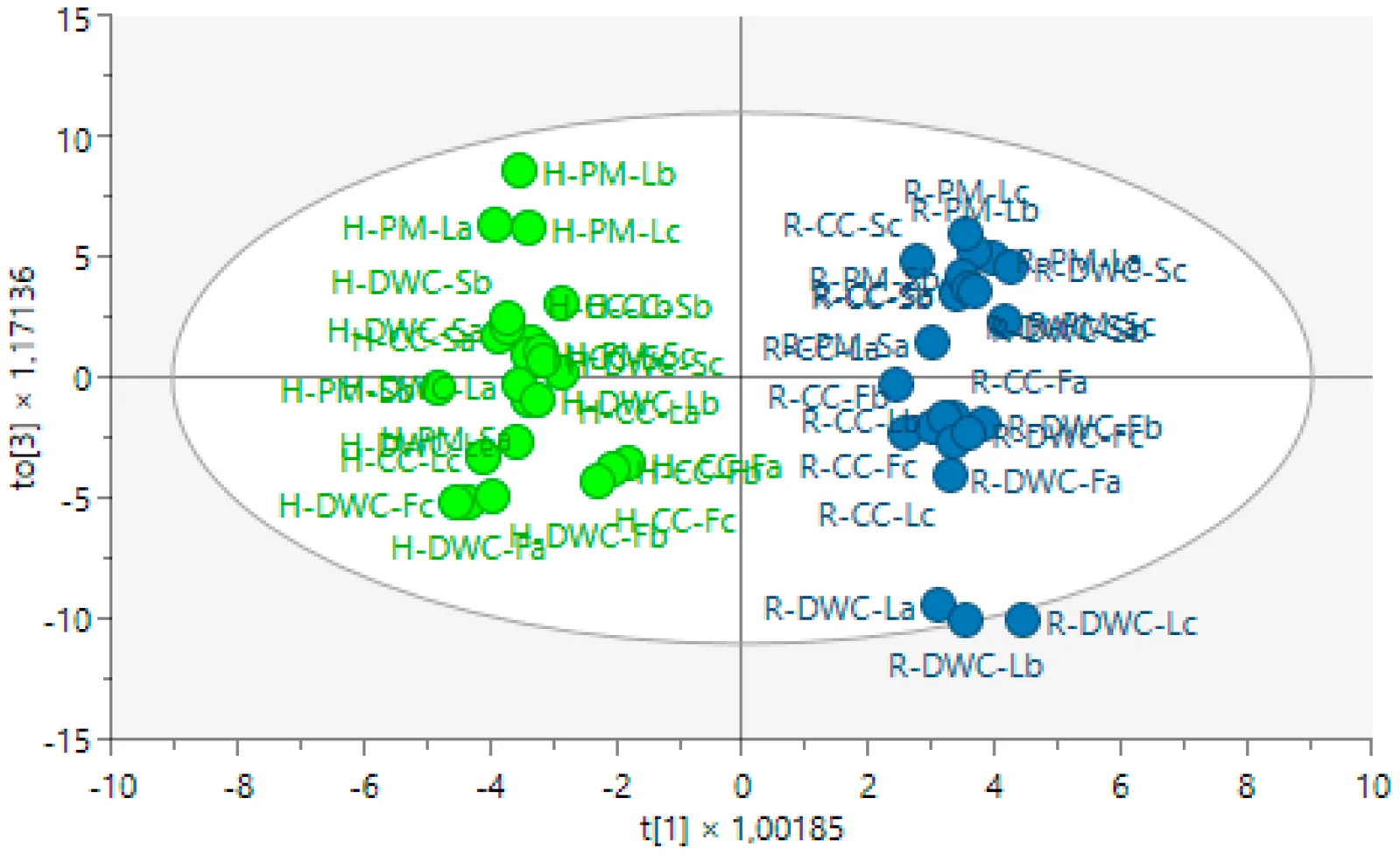
OPLS-DA score plots of tomato samples cultivated in coconut coir (CC), peat moss (PM), and deep-water culture (DWC) systems. Green and blue dots represent the Heinz and Roma cultivars, respectively.

The metabolomic differences between Heinz and Roma tomato cultivars grown in coconut coir, peat moss, and deep-water culture (DWC) systems were assessed using Principal Component Analysis (PCA) and Orthogonal Partial Least Squares Discriminant Analysis (OPLS-DA). Significant metabolic variation between samples was indicated by the PCA model (Figure 2), which showed distinct clustering patterns linked to cultivar and cultivation treatment. With R^2^X(cum) = 0.81 and Q^2^(cum) = 0.66, the model demonstrated strong explanatory and predictive power. There were two outliers that matched Roma leaf samples cultivated in DWC. The OPLS-DA model (Figure 3) showed excellent model performance (R^2^X(cum) = 0.95, R^2^Y(cum) = 0.99, and Q^2^(cum) = 0.99) and further enhanced class discrimination. Model robustness was confirmed by cross-validation, permutation testing, and CV-ANOVA, which also showed that the observed separation could not be attributed to random variation (*p* < 0.05).

The OPLS-DA multivariate statistical analysis (Figure 2) revealed a clear discrimination between the cultivars and different growth media, with only two outliers (Roma cultivar in DWC leaf a and b). The model showed a good fit and predictability in accordance with R^2^X(cum) = 0.95, R^2^Y = 0.99 (cum), and Q^2^ = 0.99 (Figure 2).

Strong explanatory and predictive performances were shown by the OPLS-DA model (R^2^X(cum) = 0.95, R^2^Y(cum) = 0.99, and Q^2^(cum) = 0.99). Cross-validation and permutation testing were used in the model validation process to assess the robustness of the model and rule out overfitting. The validation results confirmed that the observed class separation was not the result of random variation by showing that the original model maintained significantly higher predictive power than the permuted models. Additionally, CV-ANOVA showed statistical significance (*p* < 0.05), indicating the model’s dependability.

To further investigate the differences in the responses of Heinz and Roman tomato cultivars to the treatments of different growth media (coconut coir, peat moss, and deep-water culture), the results in Figure 2 depict PCA of 1H-NMR spectra of tomato samples treated with coconut coir (CC), peat moss (PM), and deep-water culture (DWC). Each point in the PCA scatter plot represents a demonstration sample. The PCA shows the response of leaves, stems, and fruits to CC, PM, and DWC. In addition, the model showed an excellent fit (R2X (cum) = 0.81) and predictive ability (Q2 (cum) = 0.66). Some samples showed negative values along PC1 and PC2, except for two outliers (R-DWC-La and R-DWC-Lb). Similarly, Figure 3 shows similar results, except for two outliers (R-DWC-Lb and R-DWC-Lc).

The metabolomic differences between Heinz and Roma tomato cultivars grown under coconut coir, peat moss, and deep-water culture (DWC) systems were assessed using Principal Component Analysis (PCA) and Orthogonal Partial Least Squares Discriminant Analysis (OPLS-DA). Different metabolomic profiles among samples were indicated by the PCA model (Figure 2), which clearly clustered according to cultivar and cultivation system (R^2^X(cum) = 0.81, Q^2^(cum) = 0.66).

The OPLS-DA model (Figure 3) showed strong discrimination between cultivars and treatments and further enhanced group separation (R^2^X(cum) = 0.95, R^2^Y(cum) = 0.99, Q^2^(cum) = 0.99). Cross-validation, permutation testing, and CV-ANOVA (*p* < 0.05) were used to validate the model, confirming its reliability and ruling out overfitting.

**Figure 4:**
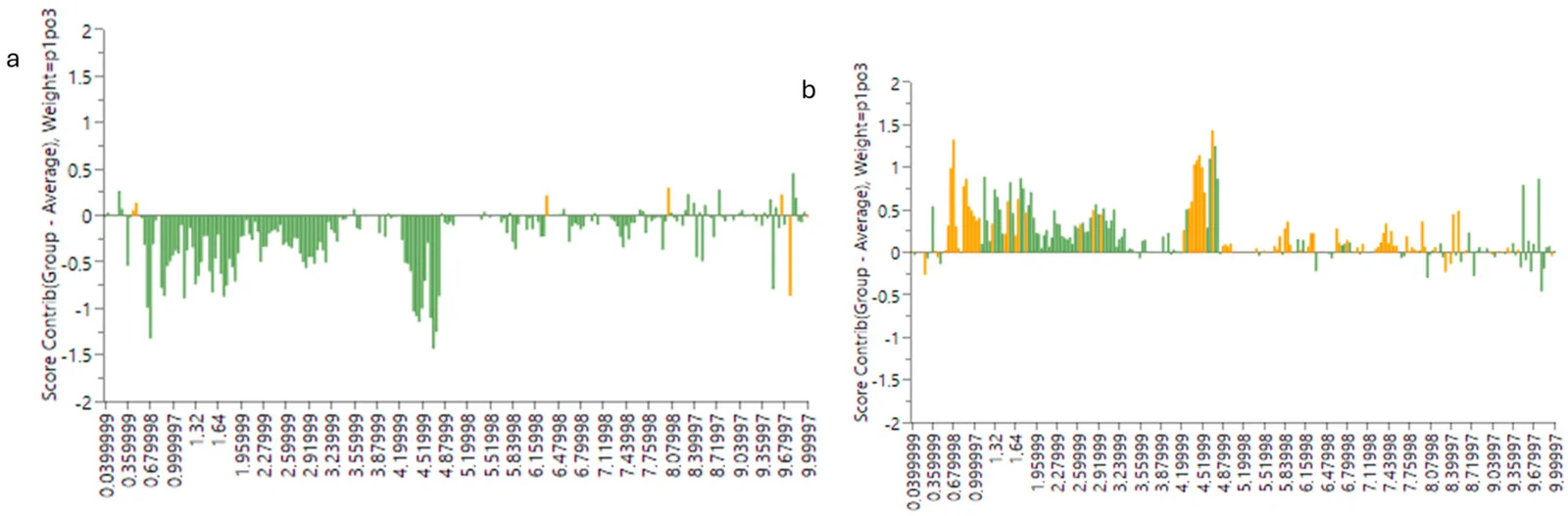
Contribution plots of tomato cultivars grown under coconut coir (CC), peat moss (PM), and deep-water culture (DWC) systems. (**a**) Heinz cultivar; (**b**) Roma cultivar.

The resulting ^1^H NMR contribution plot (Figure 4) demonstrates the variations caused by the different cultivars (Heinz and Roma). The loadings of each variable in the contribution plot (Figure 4) illustrate the chemical shifts from the metabolomic databases for metabolite identification. The contribution plot showed a clear separation between and within the cultivars, suggesting high metabolite variations between the samples. Sugars and other primary metabolites were significant contributors to the observed divergence across cultivars and treatments. The sugar area in the Heinz tomato organ cultivar appeared to differ markedly, suggesting that carbohydrate metabolism changed between the cultivation regimes.

Figure 4’s OPLS-DA contribution plots revealed the metabolites in charge of cultivar discrimination. The primary contributors were carbohydrates and associated primary metabolites in the 3.0–5.5 ppm range, suggesting that Heinz and Roma cultivars differ in their carbon metabolism and sugar accumulation.

**Figure 5:**
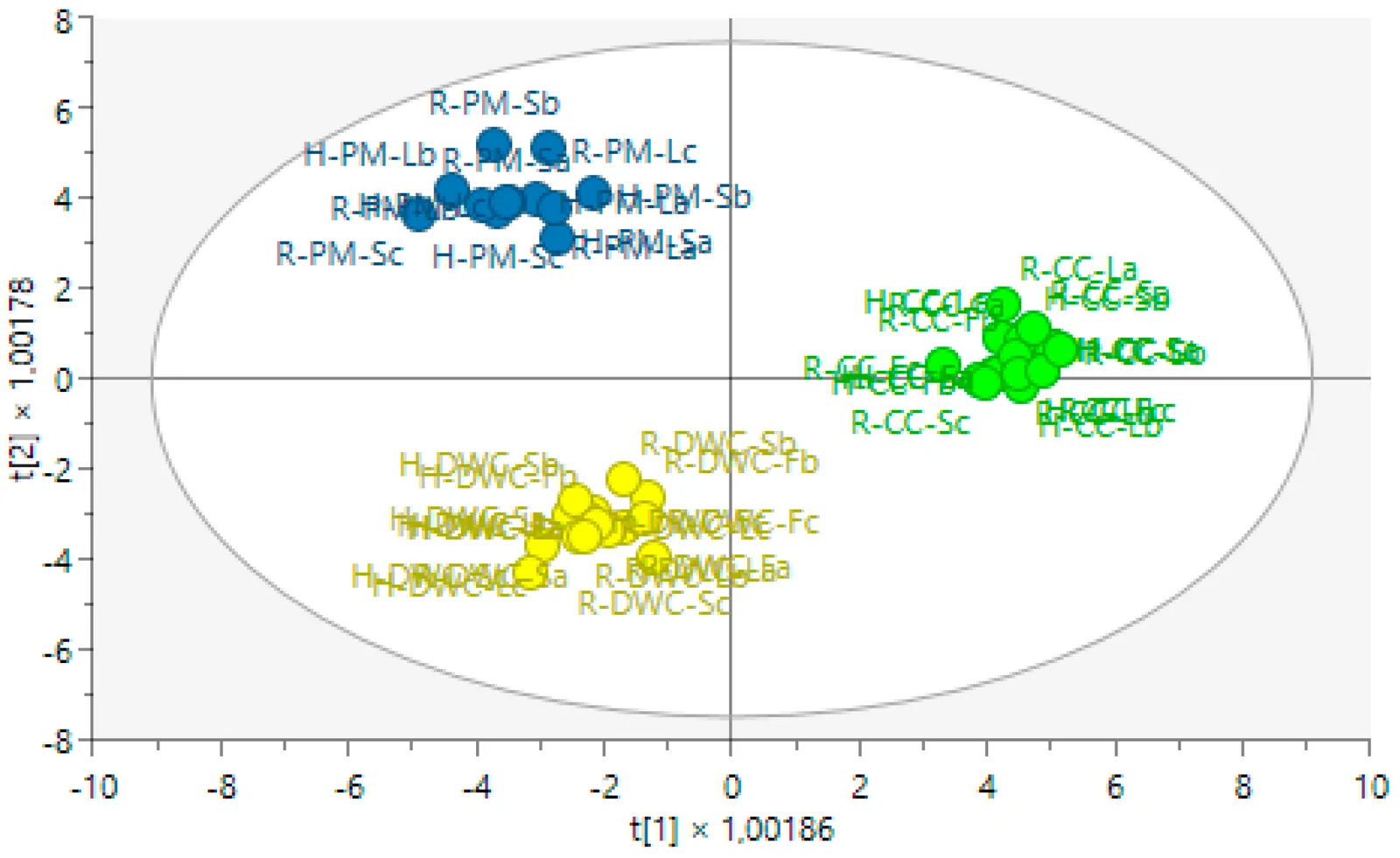
PLS-DA score plots of tomato samples cultivated under coconut coir (CC), peat moss (PM), and deep-water culture (DWC) systems. Green, blue, and yellow dots represent samples grown in CC, PM, and DWC, respectively.

**Figure 6:**
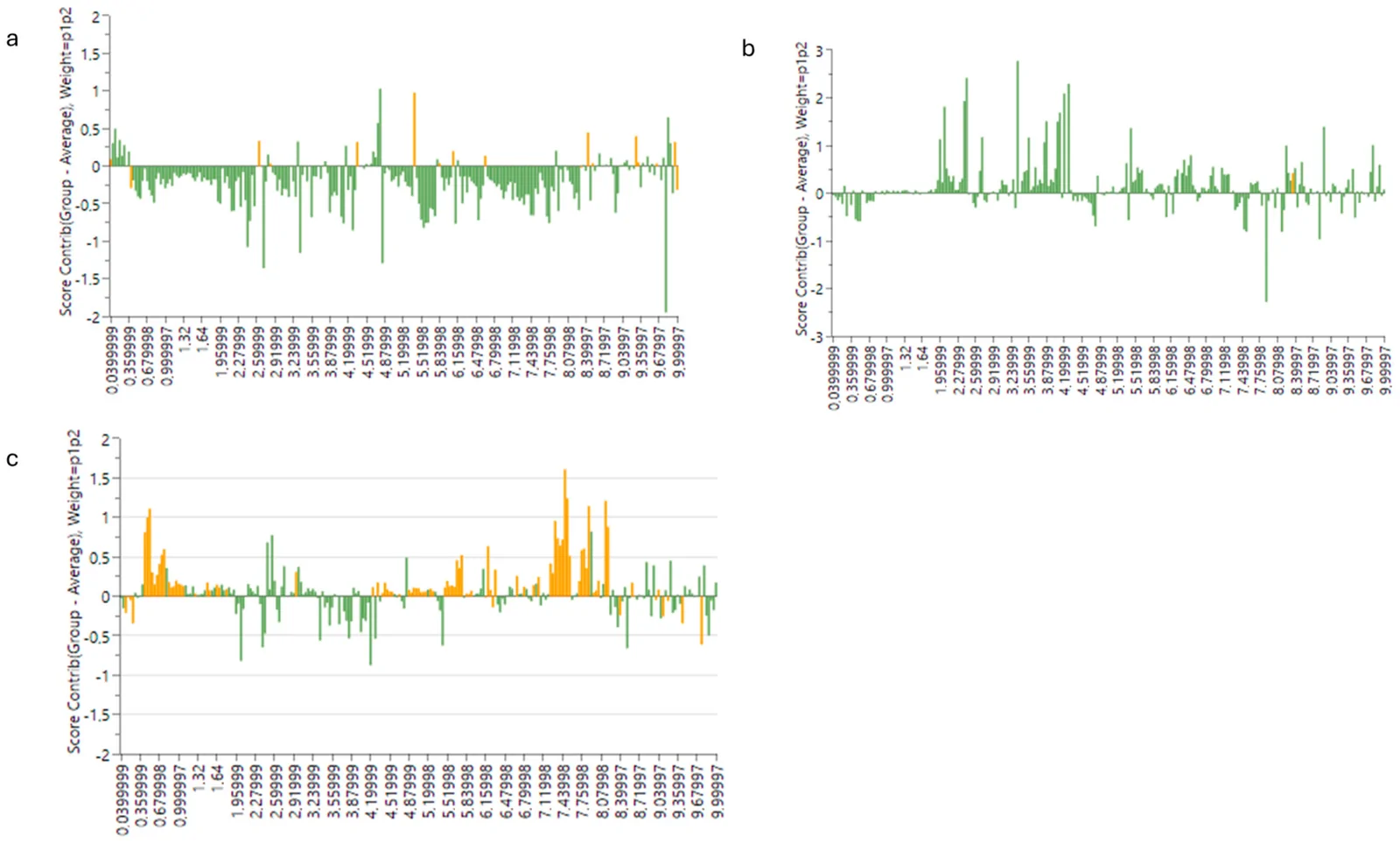
Contribution plots of tomato cultivar samples cultivated under coconut coir (CC), peat moss (PM), and deep-water culture (DWC) systems. (**a**) CC-grown samples; (**b**) PM-grown samples; (**c**) DWC-grown samples.

Tomato samples grown in coconut coir, peat moss, and DWC systems were clearly separated according to PLS-DA analysis (Figure 5), suggesting that the cultivation environment had a major impact on metabolite composition. Contribution plots (Figure 6) demonstrated that changes in sugars, organic acids, and compounds related to amino acids drove this separation, indicating the impact of cultivation conditions on central metabolic pathways.

**Figure 7:**
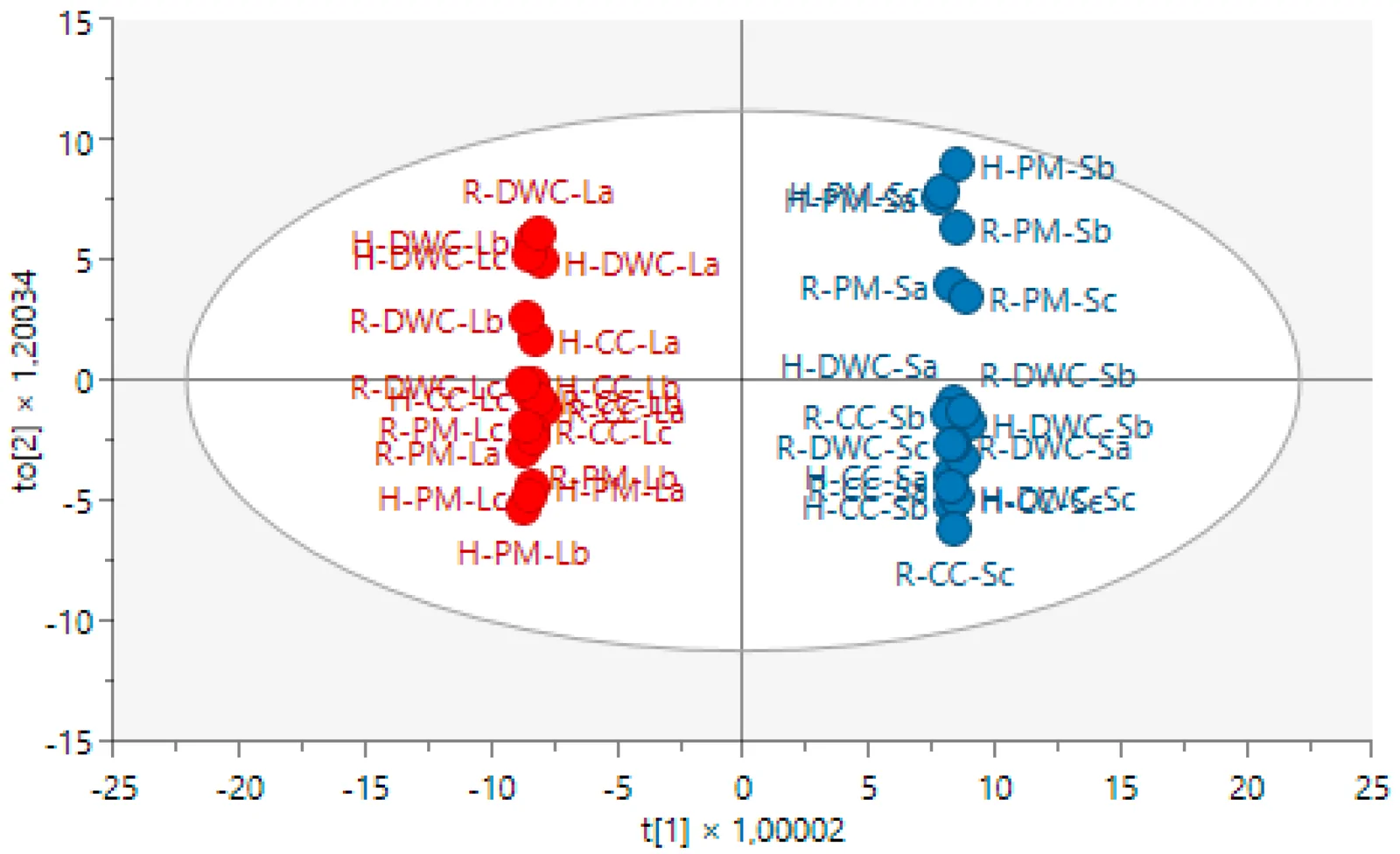
PLS-DA score plots showing the distribution of tomato samples cultivated under coconut coir (CC), peat moss (PM), and deep-water culture (DWC) systems.

**Figure 8:**
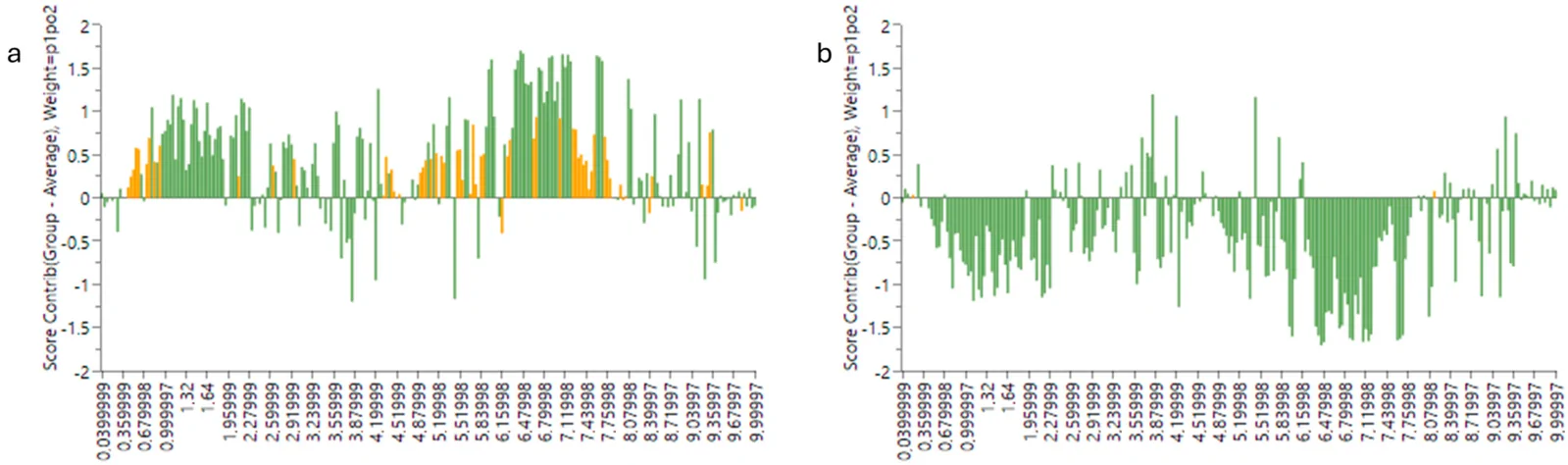
Contribution plots of tomato cultivar samples cultivated under coconut coir (CC), peat moss (PM), and deep-water culture (DWC) systems. (**a**) Heinz cultivar; (**b**) Roma cultivar.

Tomato plants’ metabolic variation was clearly dependent on both tissue and treatment, according to combined multivariate analyses (Figure 7 and Figure 8). Great changes in the metabolite composition of plant organs were indicated by the distinct clustering of fruits, leaves, and stems. Within each tissue type, treatment-dependent separation was also seen, demonstrating that the cultivation system had an impact on metabolic profiles at the organ and whole-plant levels.

Growth media significantly influence tomato metabolites. As shown in Table 3, the Heinz tomato cultivar grown in peat moss (PM) exhibited the highest allantoin concentration, at 14.52 mM. This was followed by tomatoes cultivated in deep water culture (DWC), while the lowest allantoin concentration, measured at 7.45 mM, occurred when tomatoes were grown in coconut coir. Additionally, the cultivar itself played a crucial role, with the Roma cultivar displaying the highest allantoin concentration when cultivated in peat moss.

Coconut coir exhibited the highest concentration of erythritol at 6.13 mM. Peat moss followed closely for the Heinz cultivars, recording a concentration of 4.70 mM, while deep water culture had the lowest concentration at 2.70 mM. In contrast, for the Roma cultivar tomatoes, those grown on coconut coir and peat moss showed no significant difference, with concentrations of 3.70 mM and 3.72 mM, respectively. Notably, the highest concentration was observed in tomatoes grown under deep water culture, achieving 7.20 mM, which surpasses the concentration recorded for the Heinz cultivar.

The metabolites found in this study are primarily linked to energy production, antioxidant defense, stress-response pathways, and carbohydrate metabolism. Galactose, mannitol, trehalose, erythritol, and xylitol are examples of sugars and sugar alcohols that show variations in osmotic regulation and carbon allocation between treatments. Ascorbate is an important antioxidant that protects against oxidative stress, and 2-phosphoglycerate is a crucial glycolytic intermediate that signals changes in energy metabolism. Overall, these findings imply that important biochemical pathways connected to growth, stress tolerance, and fruit quality are influenced by both cultivation system and genotype.

## 4. Discussion

The current study showed that the cultivation system and cultivar genotype had a significant impact on the metabolomic profiles of tomato fruits. Fruit biochemical composition is largely determined by genetic background, as evidenced by the obvious metabolic differences between the Heinz and Roma cultivars, even though they were grown in the same greenhouse. The accumulation of primary metabolites may have been influenced by genotype-driven variations in metabolic regulation, according to the separation seen in the PCA model (Figure 2). Genetic factors have been demonstrated to affect sugar accumulation, organic acid content, and overall fruit quality attributes in previous reports of cultivar-dependent metabolic variation [13,14]. These results show that genetic characteristics and breeding background may be important in determining how tomato plants distribute carbon and nutrients during fruit development.

The OPLS-DA and PLS-DA models, which showed high R^2^ and Q^2^ values, indicating strong explanatory and predictive capabilities, further validated the discrimination between cultivars and cultivation treatments. The observed metabolic differences were biologically significant rather than the result of random variation, as evidenced by the consistency of clustering patterns across multivariate models. According to related research, combining chemometric techniques with 1H-NMR metabolomics offers a dependable platform for identifying minute metabolic alterations linked to genotype and environmental factors [5,15]. The robustness of the analytical approach for characterizing metabolomic responses in tomato production systems is thus supported by the strong model performance found in this study.

Carbohydrates were one of the main metabolites responsible for sample discrimination, according to the contribution plots, especially in the spectral range of 3.0–5.5 ppm. Glucose, fructose, sucrose, and sugar alcohols—all significant factors influencing fruit taste, sweetness, and market quality—were the main causes of the observed variations [16,17]. The Heinz cultivar’s higher variation indicates that this genotype may be more sensitive to cultivation conditions in terms of carbon metabolism and assimilate partitioning. This result is in line with earlier studies that demonstrate how environmental factors affect photosynthate allocation and sugar accumulation during fruit development, which in turn affects fruit quality characteristics [6,17]. The findings therefore suggest that the efficiency with which carbon resources are transformed into fruit metabolites may be influenced by interactions between cultivation system and genotype.

The metabolite composition was also significantly influenced by the cultivation system, as demonstrated by the distinct separation of samples grown in DWC, peat moss, and coconut coir systems. In contrast to substrate-based systems, hydroponic conditions may have changed metabolic pathways, as evidenced by the distinct clustering of DWC-grown plants. Nutrient uptake efficiency and metabolic regulation may be impacted by DWC’s continuous provision of water and nutrients while altering root-zone oxygen availability [8]. Due to variations in nutrient dynamics and root environmental conditions, hydroponic cultivation can cause major alterations in primary and secondary metabolism, according to earlier research [7,8]. The current research offers support to the idea that cultivation systems have an impact on both plant growth and the biochemical processes that determine fruit quality and composition.

The treatment contribution plots, which identified amino acids, organic acids, and antioxidants as important discriminating metabolites, offered additional proof of cultivation-system effects. Respiration, redox regulation, and stress adaptation all depend on metabolites like citric acid, malic acid, and ascorbate [17]. Differential physiological reactions to nutrient availability and root-zone conditions may be indicated by variations in these compounds’ abundance across treatments. Increased organic acid accumulation, for instance, might be a sign of changes in energy metabolism, while variations in antioxidant levels might be a sign of different reactions to oxidative stress. Plants exposed to different cultivation environments have been shown to undergo similar metabolic adjustments, with changes in primary metabolism and stress-response pathways brought on by changes in nutrient supply and environmental conditions [18,19]. These results show that metabolic processes linked to plant health and fruit nutritional quality can be directly influenced by cultivation practices.

**Figure 9:**
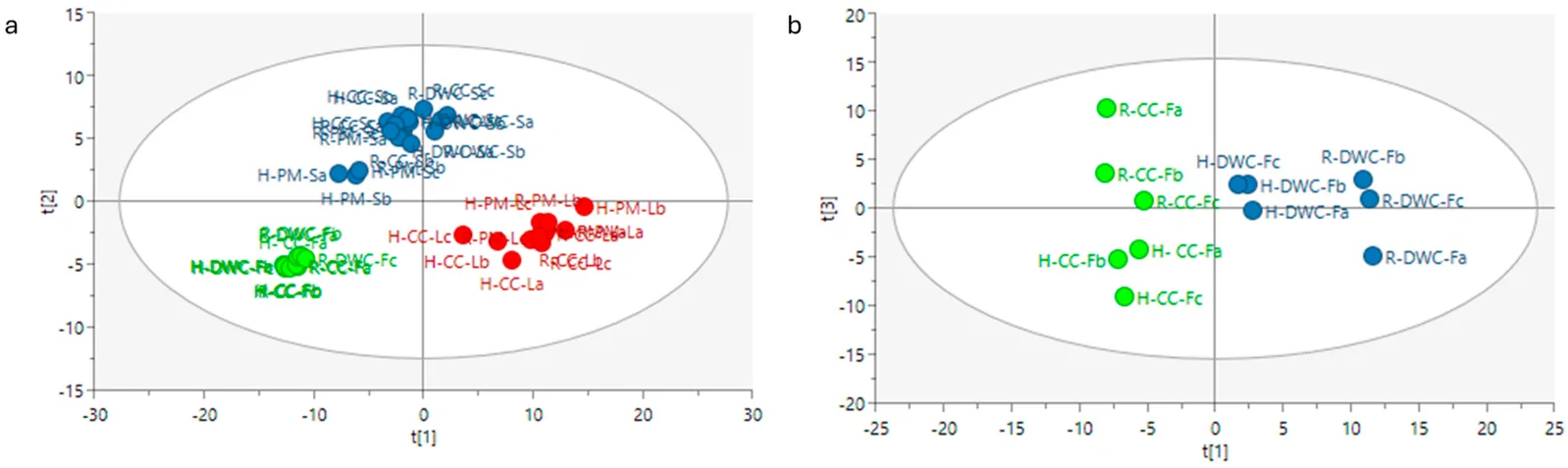
(**a**) is the OPLS-DA score plot of stem, fruits and leaves tomato samples treated with coconut coir (CC), peat moss (PM), and deep-water culture (DWC). (**b**) illustrates the PLS-DA of fruits treated with coconut coir (CC); peat moss (PM), and deep-water culture (DWC). The green dots represent the tomato samples treated Shows how tomato tissues differ metabolically depending on the organ. There are strong tissue-specific metabolite accumulation patterns when leaves, stems, and fruits are clearly separated. Additionally, fruit samples cultivated in DWC and coconut coir conditions formed unique clusters, indicating that fruit metabolite composition is directly influenced by the root-zone environment. These variations suggest that cultivation systems may modify metabolic pathways linked to the development of fruit quality, energy metabolism, and carbon allocation.

Fruits, leaves, and stems were found to have different tissue-specific metabolic profiles. Fruits are especially sensitive to changes in cultivation conditions, as evidenced by the greater treatment-dependent variation found in fruit tissues. Fruits accumulate a variety of metabolites linked to flavor, nutritional value, and reproductive development because they are the main sink organs. Fruit samples from DWC and coconut coir were found to be separated (Figure 9b), indicating that root-zone conditions affect metabolite transport and allocation to developing fruits. Previous metabolomic studies have reported similar tissue-specific responses, showing that different plant organs have distinct metabolic adaptations due to their specialized physiological functions [5,20]. These results demonstrate how essential it is to take tissue-specific reactions into account when assessing how cultivation methods affect plant metabolism.

Different clustering patterns showed cultivar-specific metabolic adaptability even though both cultivars reacted to changes in growth media. Heinz seemed to be more metabolically responsive to cultivation conditions than Roma, indicating that the genotypes differed in their physiological plasticity. Genetic variations controlling the efficiency of nutrient uptake, carbon partitioning, and stress-response mechanisms may be the source of this variation. Similar genotype-dependent reactions have been documented in tomatoes and other horticultural crops, where cultivars vary in their ability to modify metabolic pathways in response to environmental changes. These results highlight the significance of choosing cultivar–cultivation system combinations that maximize fruit quality and productivity from a practical standpoint. The findings also indicate that adding metabolomic data into cultivation management techniques may help create a tomato production system that is more productive and quality-focused.

## 5. Conclusions

This study showed that metabolomic analysis based on ^1^H-NMR is a useful method for describing the biochemical changes in tomato cultivars grown in various cultivation systems. The Heinz and Roma cultivars, as well as the three growth media—coconut coir, peat moss, and deep-water culture—all showed distinct metabolic differences. Research has shown that important metabolite groups, especially sugars, organic acids, and amino acids, which are critical for fruit quality and plant physiological processes, are greatly influenced by the cultivation system used. Compared to substrate-based systems, deep-water culture generates a different metabolic profile, indicating that root-zone conditions and ongoing nutrient availability are important factors influencing plant metabolism.

The potential of metabolomics in biomarker discovery was demonstrated by the identification of metabolites responsible for cultivar discrimination and treatment. Future breeding and cultivation techniques may use these biomarkers to improve fruit quality, nutritional content, and system adaptability.

In summary, the metabolite composition of tomato is significantly influenced by both genotype and cultivation technique. Developing optimized and sustainable tomato production systems will require expanding this research to include more cultivars and environmental factors, especially given the growing popularity of hydroponic technologies.

## Figures and Tables

**Table 1:** Factorial design of cultivar × growth system treatments.

Growth System	Heinz	Roma
Coconut coir	T1	T2
Peat moss (control)	T3	T4
Deep-water culture (DWC)	T5	T6

**Table 2:** Physicochemical characteristics of the growth media and nutrient composition of the nutrient solution used in this study.

Growth Medium/Nutrient Solution	Key Characteristics	Nutrient Composition
Coconut coir	Organic fibrous substrate; high water retention and aeration	Very low inherent N, P, K
Peat moss (control)	Porous organic substrate; high moisture retention; acidic pH	Low inherent N, P, K
Nitrosol Organiksol (DWC solution)	Liquid organic-based fertilizer	N (80 g kg^−1^), P (20 g kg^−1^), K (58 g kg^−1^), Mg (7 g kg^−1^), Ca (6 g kg^−1^), S (4 g kg^−1^); Fe (60 mg kg^−1^), Cu (1 mg kg^−1^), Zn (1 mg kg^−1^), Mn (40 mg kg^−1^), B (23 mg kg^−1^), Mo (15 mg kg^−1^); GA_3_ (3 mg kg^−1^)

**Table 3:** Annotation of metabolites identified in tomato cultivars (Heinz and Roma) grown under coconut coir (CC), peat moss (PM), and deep-water culture (DWC) systems.

Sample	Compound Name	Concentration (mM)	NMR Chemical Shifts and Multiplicity	Chenomx 12.0
Heinz				
Coconut coir (CC)	1,3-Dimethylurate	1.62	δ_H_: 3.29 (s), 3.45 (s)	3.31, 3.45
	Acetamide	0.39	δ_H_: 1.97 (s)	1.98
	Allantoin	7.45	δ_H_: 5.37 (s)	5.39
	Arabinitol	1.20	δ_H_: 3.55 (m)	3.57
	Ascorbate	0.34	δH: 4.51	4.53
	Cellobiose	2.63	δ_H_: 4.66 (d), 3.96 (m)	4.45, 3.96
	Erythritol	6.13	δ_H_: 3.74 (m)	3.75
	Fructose	2.20	δH:3.88 (dd)	3.89
	Glucose	1.23	δ_H_: 4.64 (dd), 5.24 (d)	5.24
	Lactose	1.21	δ_H_: 3.93 (m)	3.89
	Maltose	15.53	δ_H_: 5.38 (d)	5.37
	Mannitol	0.94	δ_H_: 3.88 (dd)	3.87
	Mannose	2.35	δ_H_: 3.94 (d)	3.94
	Ribose	5.82	δ_H_: 5.63 (d)	5.36
	Xylitol	1.71	δ_H_: 3.71 (m)	3.73
Peat moss (PM)	1,3-Dimethylurate	0.30	δ_H_: 3.45 (s)	3.45
	Acetamide	0.24	δ_H_: 1.99 (s)	1.98
	Allantoin	14.52	δ_H_: 5.39 (s)	5.38
	Arabinitol	1.16	δ_H_: 3.56 (dd)	3.57
	Ascorbate	0.37	δ_H_: 4.51 (dd)	4.51
	Cellobiose	3.31	δ_H_: 4.66 (dd)	4.67
	Erythritol	4.70	δ_H_: 3.76 (m)	3.77
	Fructose	2.88	δ_H_: 3.88 (dd)	3.89
	Glucose	1.52	δ_H_: 4.63 (d)	4.64
	Lactose	1.81	δ_H_: 3.92 (m)	3.93
	Maltose	24.24	δ_H_: 5.38 (dd)	5.39
	Mannitol	1.15	δ_H_: 3.87 (dd)	3.88
	Mannose	3.22	δ_H_: 3.93 (m)	3.94
	Ribose	1.72	δ_H_: 5.25 (d)	5.25
	Xylitol	2.44	δ_H_: 3.62 (m)	3.63
Deep-water culture (DWC)	1,3-Dimethylurate	0.40	δ_H_: 3.44 (s)	3.45
	Acetamide	0.31	δ_H_: 2.00 (s)	2.00
	Allantoin	13.87	δ_H_: 5.39 (s)	5.39
	Arabinitol	1.86	δ_H_: 3.57 (dd)	5.58
	Ascorbate	0.55	δ_H_: 4.50 (d)	4.51
	Cellobiose	3.18	δ_H_: 4.65 (d)	4.66
	Erythritol	2.70	δ_H_: 3.76 (m)	3.77
	Fructose	3.07	δ_H_: 3.90 (dd)	3.90
	Glucose	0.35	δ_H_: 5.24 (d)	5.23
	Lactose	1.55	δ_H_: 4.46 (dd)	4.45
	Maltose	20.41	δ_H_: 3.42 (dd)	3.41
	Mannitol	1.87	δ_H_: 3.67 (dd)	3.66
	Mannose	4.92	δ_H_: 3.95 (m)	3.94
	Ribose	4.34	δ_H_: 4.92 (dd)	4.91
	Xylitol	3.03	δ_H_: 3.73 (dd)	3.70
Roma				
Coconut coir (CC)	1,3-Dimethylurate	0.15	δ_H_: 3.45 (s)	3.44
	Acetamide	0.35	δ_H_: 1.99 (s)	2.00
	Allantoin	8.34	δ_H_: 5.38 (s)	5.37
	Arabinitol	2.86	δ_H_: 3.57 (dd)	5.58
	Ascorbate	0.29	δ_H_: 4.51 (d)	4.51
	Cellobiose	1.40	δ_H_: 4.66 (dd)	4.66
	Erythritol	3.70	δ_H_: 3.76 (m)	3.77
	Fructose	1.84	δ_H_: 3.89 (dd)	3.88
	Glucose	2.88	δ_H_: 3.90 (dd)	3.84
	Lactose	6.31	δ_H_: 4.66 (d)	4.44
	Maltose	14.42	δ_H_: 5.38 (dd)	5.37
	Mannitol	0.70	δ_H_: 3.88 (dd)	3.87
	Mannose	4.72	δ_H_: 4.89 (d)	4.88
	Ribose	0.86	δ_H_: 5.24 (m)	5.24
	Xylitol	0.73	δ_H_: 3.66 (m)	3.65
Peat moss (PM)	1,3-Dimethylurate	0.28	δ_H_: 3.45 (s)	3.44
	Acetamide	0.41	δ_H_: 2.00 (s)	1.99
	Allantoin	19.78	δ_H_: 5.39 (s)	5.38
	Arabinitol	1.48	δ_H_: 3.58 (dd)	3.57
	Ascorbate	0.28	δ_H_: 4.51 (d)	4.49
	Cellobiose	1.26	δ_H_: 4.50 (d)	4.50
	Erythritol	3.72	δ_H_: 3.76 (m)	3.77
	Fructose	3.99	δ_H_: 3.69 (m)	3.67
	Glucose	12.61	δ_H_: 3.40 (dd)	3.39
	Lactose	8.67	δ_H_: 4.66 (m)	4.53
	Maltose	6.23	δ_H_: 3.63 (m)	3.59
	Mannitol	1.81	δ_H_: 3.88 (dd)	3.77
	Mannose	6.83	δ_H_: 4.89 (d)	4.91
	Ribose	5.41	δ_H_: 4.92 (dd)	4.90
	Xylitol	6.17	δ_H_: 3.74 (dd)	3.71
Deep-water culture (DWC)	1,3-Dimethylurate	0.54	δ_H_: 3.57 (s)	3.55
	Acetamide	0.81	δ_H_: 1.98 (s)	1.99
	Allantoin	4.17	δ_H_: 5.39 (s)	5.38
	Arabinitol	1.48	δ_H_: 3.58 (dd)	3.57
	Ascorbate	0.28	δ_H_: 4.51 (d)	4.49
	Cellobiose	15.27	δ_H_: 3.58 (d)	3.62
	Erythritol	7.52	δ_H_: 3.77 (dd)	3.79
	Fructose	6.52	δ_H_: 3.89 (dd)	3.88
	Glucose	8.70	δ_H_: 3.90 (d)	3.89
	Lactose	9.19	δ_H_: 3.58 (dd)	3.56
	Maltose	7.37	δ_H_: 5.39 (dd)	5.37
	Mannitol	3.41	δ_H_: 3.78 (dd)	3.75
	Mannose	8.15	δ_H_: 4.89 (s)	4.87
	Ribose	16.33	δ_H_: 3.88 (d)	3.85
	Xylitol	4.58	δ_H_: 3.63 (m)	3.64

## Data Availability

The data supporting the findings of this study are available from the corresponding author, U.V.O., upon reasonable request.
